# Management of persistent soft tissue residuals exhibiting an imaging paradox (^123I-MIBG-negative/^68Ga-DOTA-NOC-positive) in high-risk neuroblastoma following immunotherapy: a case report

**DOI:** 10.3389/fimmu.2026.1867416

**Published:** 2026-07-01

**Authors:** Si-jia He, Ju Gao, Guo-qian He, Xia Guo, Xiao-yu Jing

**Affiliations:** 1 Department of Pediatrics, West China Second University Hospital, Sichuan University, Chengdu, Sichuan, China; 2 Key Laboratory of Birth Defects and Related Diseases of Women and Children, Ministry of Education, West China Second University Hospital, Sichuan University, Chengdu, Sichuan, China

**Keywords:** 123I-MIBG, 68Ga-DOTA-NOC PET/CT, anti-GD2 immunotherapy, high-risk neuroblastoma, multimodal imaging, residual disease

## Abstract

Managing persistent soft tissue residual disease in high-risk neuroblastoma (HR-NB) remains a clinical challenge, particularly when conventional response assessments suggest remission but residual lesions persist. Determining whether such lesions represent inactive treatment-related changes or viable tumor has important implications for subsequent management. We report a 4-year-and-11-month-old girl with HR-NB who received multimodal therapy, including induction chemotherapy, surgery, radiotherapy, autologous stem cell transplantation, and seven cycles of dinutuximab beta-based immunotherapy. Following treatment, standard evaluations demonstrated complete bone marrow clearance and a ^123I-MIBG Curie score of 0, suggesting an excellent systemic response. However, a persistent paravertebral residual lesion remained detectable on imaging. Notably, ^68Ga-DOTA-NOC PET/CT demonstrated persistent tracer uptake despite negative ^123I-MIBG findings, creating an imaging discordance that raised concern for residual viable disease. Subsequent “third-look” surgical resection confirmed viable neuroblastoma within the lesion. Pathological examination revealed a markedly increased Ki-67 labeling index compared with the post-induction specimen (50% versus 8%), indicating persistent proliferative activity and suggesting biological heterogeneity within the residual lesion. This case highlights the diagnostic and therapeutic challenges posed by persistent soft tissue residual lesions after immunotherapy. The discordance between ^123I-MIBG and ^68Ga-DOTA-NOC imaging suggests that conventional imaging alone may not fully characterize selected residual lesions. In patients with persistent masses and discordant imaging findings, multimodal functional imaging may provide complementary information to guide clinical decision-making. The favorable outcome observed in this patient, who remains in sustained remission following surgical resection without additional antitumor therapy, supports consideration of an integrated management strategy combining multimodal imaging assessment and appropriately timed surgical intervention for selected cases of persistent residual disease.

## Introduction

High-risk neuroblastoma (HR-NB) is an aggressive pediatric malignancy associated with substantial biological heterogeneity and a high risk of relapse despite advances in multimodal therapy. Current treatment strategies typically incorporate intensive induction chemotherapy, surgical resection, radiotherapy, autologous stem cell transplantation, and anti-GD2 immunotherapy, resulting in significant improvements in survival over recent decades (1). Nevertheless, the management of residual disease remains a major clinical challenge, particularly in patients with persistent soft tissue lesions following completion of frontline treatment (2).

Anti-GD2 immunotherapy has become an essential component of maintenance therapy for HR-NB and has demonstrated efficacy in reducing minimal residual disease and improving long-term outcomes (3). However, residual masses frequently persist after treatment, and their biological significance may be difficult to determine. While some lesions represent treatment-related maturation, fibrosis, or necrosis, others may harbor viable tumor cells despite the absence of overt clinical or radiological progression (4). Distinguishing between these possibilities is important because it directly influences subsequent management decisions, including surveillance, additional therapy, or surgical intervention.

Functional imaging plays a central role in the evaluation of residual neuroblastoma. ^123I-MIBG scintigraphy remains the standard imaging modality for disease assessment and response evaluation. However, the interpretation of persistent residual lesions can occasionally be challenging, particularly when imaging findings are discordant or when residual masses remain stable despite otherwise favorable treatment responses. In such situations, additional imaging modalities may provide complementary information regarding residual disease activity and help guide clinical decision-making.

Herein, we report a child with HR-NB who developed persistent soft tissue residual disease following multimodal treatment and anti-GD2 immunotherapy. The residual lesion demonstrated an unusual imaging pattern, characterized by negative ^123I-MIBG findings but persistent uptake on ^68Ga-DOTA-NOC PET/CT. Subsequent surgical resection confirmed the presence of viable residual tumor and revealed a higher Ki-67 labeling index compared with previous pathological specimens. This case highlights the diagnostic and management challenges posed by persistent residual lesions with discordant imaging findings and illustrates the potential value of integrating multimodal imaging and surgical assessment in selected patients.

## Case presentation

### Initial presentation and baseline profiling

A 4-year-and-11-month-old female patient initially presented to our institution in March 2023 with primary symptoms of persistent abdominal pain and fever. Physical examination upon admission revealed a bloating and mild tenderness, with no focal neurological deficits. Baseline laboratory investigations revealed an elevated neuron-specific enolase (NSE) of 126 ng/mL(reference range<16.3ng/mL) and increased catecholamine metabolites: dopamine (4.79 nmol/L, reference range<0.20 nmol/L), normetanephrine (2.53 nmol/L, reference range<0.60nmol/L), and 3-methoxytyramine (153.82 pg/mL, reference range<15pg/mL). The complete blood count indicated moderate anemia (WBC: 6.5×10^9^/L, LYMPH%: 17%, NEUT%: 62%, HB: 76 g/L, HCT: 22.3%, MCV: 83.8 fl, MCH: 28.6 pg, MCHC: 341 g/L, PLT: 440×10^9^/L) with normal CRP (<0.5 mg/L).

Initial contrast-enhanced CT (February 21, 2023) revealed extensive, calcified retroperitoneal lymphadenopathy. A baseline PET/CT (March 9, 2023) demonstrated widespread hypermetabolic disease against a liver background SUVmax of 1.24 (mean 0.92). Active tumors were identified in the left supraclavicular/thoracic lymph nodes (SUVmax 2.79), abdominal and gluteal soft tissues (SUVmax 6.26), left kidney (SUVmax 7.97), and multiple bone marrow (BM) lesions (SUVmax 34.33).

A core biopsy (March 2, 2023) confirmed a malignant neurogenic tumor (partially differentiated neuroblastoma). The comprehensive immunohistochemistry (IHC) profile was: CgA(+), Syn(+), CD56(+), CK(Pan)(-), CD99(-), NKX2.2(-), INI1(-), Desmin(-), Myogenin(-), SALL4(-), WT1(-), and C-MYC amplification(-). Notably, the tumor exhibited a highly aggressive Ki-67 index of 80%. Bilateral BM aspirates were MRD-positive with increased MYCN copy numbers. Whole-genome microarray analysis (sampled March 22, 2023) revealed a hyperdiploid karyotype with mosaic loss in 1p36.33-p35.3 and 11q22.3-q25, alongside mosaic copy-neutral loss of heterozygosity (CN-LOH) in 11q13.4-q22.3, definitively stratifying the patient as high-risk.

### Induction therapy, secondary surgery, and consolidation

Treatment commenced on March 20, 2023, strictly following the CCCG-NB-2021 protocol. Following 4 induction cycles, a comprehensive reassessment (May 5, 2023) demonstrated a partial response: CT scans showed a 76% volumetric reduction of the presacral mass, alongside shrinkage of adjacent nodes. Serum NSE dropped to 27.8 ng/mL. BM evaluation (May 3) showed clearance on the right side, though the left side remained MRD-positive (9.31%).

A secondary surgical resection of the primary and pelvic lesions was performed on June 19, 2023. Postoperative pathology confirmed poorly differentiated neuroblastoma with lymph node metastasis. The IHC profile [CgA(+), CD56(+), Phox2b(+), Syn(+), CK(-), MYCN(weak +), C-MYC(weak +)] revealed a therapy-induced plunge in the Ki-67 index to approximately 8%. Following Cycle 5, NSE further normalized to 11.9 ng/mL (July 1, 2023), and CT indicated osteosclerotic changes in multiple bones, consistent with healing metastases.

Following 6 chemotherapy cycles, a critical reassessment PET/CT (August 23, 2023) identified scattered punctate high-density shadows in the previously active retroperitoneal and gluteal regions. Crucially, these lesions exhibited no obvious hypermetabolism, indicating low metabolic activity of tumor. Targeted tumor-bed radiotherapy (September 6, 2023) was then delivered: 1.8 Gy/fx bid (left psoas), 1.5 Gy/fx bid (retroperitoneum), and 1.1 Gy/fx bid (right kidney).

### Rationale and execution of GD2 maintenance immunotherapy

Post-radiotherapy, the patient met all criteria for Complete Response (CR) under the CCCG-NB-2021 guidelines: primary residual <10 mm, metabolically inactive on PET/CT, and bilateral BM MRD-negative. Based on the CCCG-NB-2021 protocol, maintenance immunotherapy was initiated.

To exert sustained immunological pressure against circulating MRD and dormant clones, the patient received a standardized immunomodulatory triad: Dinutuximab beta (100 mg/m² per cycle via 10-day continuous IV infusion) + Granulocyte-Macrophage Colony-Stimulating Factor (GM-CSF) + 13-cis-Retinoic Acid (13-cis-RA). Seven complete cycles were administered between October 17, 2023, and May 24, 2024.

To mitigate GD2-induced neurotoxicity, a strict prophylactic triple-analgesia regimen was implemented: (1) Non-opioid Ibuprofen; (2) Gabapentin (initiated 3 days prior at 10 mg/kg/day, escalating to 2×10 mg/kg/day during infusion); and (3) Continuous IV Morphine (initiated 2 hours prior at 0.02–0.05 mg/kg/h, maintained at 0.03 mg/kg/h). Hypersensitivity and emesis were managed with diphenhydramine and 5-HT3 antagonists. Regarding treatment tolerability, the administration of Dinutuximab beta was generally manageable. During the immunotherapy cycles, the patient experienced expected adverse effects including grade 2 neuropathic pain and mild fever. These symptoms were effectively managed with standard supportive care, including intravenous analgesics and antipyretics. No dose-limiting toxicities, severe capillary leak syndrome, or anaphylaxis occurred, allowing the successful completion of the planned immunotherapy regimen.

### The imaging paradox and “third-look” surgery

**Figure 1:**
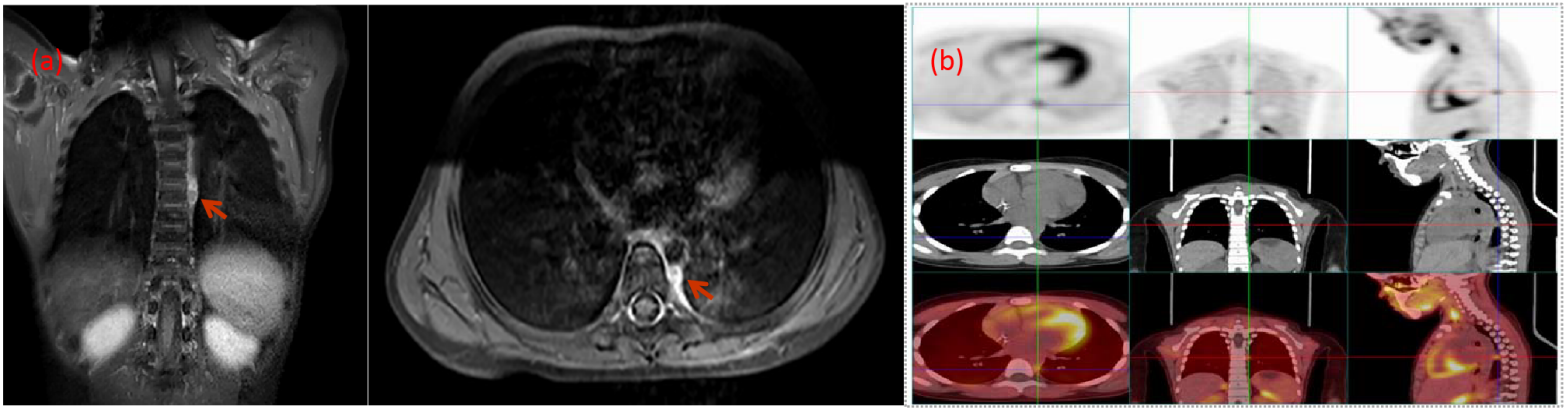
These images were acquired in July 2024 to September 2024 following the completion of consolidation and anti-GD2 immunotherapy (Dinutuximab beta). Prior to this scan, standard ^123I-MIBG scintigraphy was entirely negative. **(A)** Contrast-enhanced chest MRI (coronal and axial views) demonstrates persistent thickening of the paravertebral soft tissue on the left side, spanning thoracic vertebrae T3 to T9 (red arrows). **(B)** Given the structural remnants and the negative MIBG result, SSTR-targeted ^68Ga-DOTA-NOC PET/CT was performed to evaluate tumor viability. The scan reveals a focal nodular lesion with slightly increased SSTR expression on the left margin of the T7 vertebra (measuring approximately 0.5×0.5cm, SUVmax​=3.46).

Routine CT surveillance during immunotherapy (March 4, 2024) demonstrated an enlarged thymus (3.6 × 3.2 cm), which showed mild interval fluctuation following treatment (4.3 × 2.8 cm on July 4, 2024). Three months after completion of immunotherapy, conventional ^123I-MIBG scintigraphy demonstrated no abnormal tracer uptake, corresponding to a Curie score of 0 and suggesting complete metabolic remission. Despite these favorable findings, serial structural imaging (CT and MRI) continued to demonstrate a persistent paravertebral soft tissue lesion without significant interval reduction in size. The clinical significance of this residual mass remained uncertain, as post-treatment residual lesions may represent either inactive treatment-related changes or persistent viable tumor. Given the ongoing diagnostic uncertainty and the potential implications for further management, ^68Ga-DOTA-NOC PET/CT was performed as a complementary imaging modality. Notably, ^68Ga-DOTA-NOC PET/CT demonstrated persistent tracer uptake within a left paravertebral nodule at the T7 level measuring approximately 0.5 × 0.5 cm, with mild somatostatin receptor expression (SUVmax 3.46) (**Figure 1**). This finding was discordant with the negative ^123I-MIBG study and raised concern for residual biologically active disease.

To definitively manage this ^123I-MIBG-negative/DOTA-NOC-positive remnant, a third thoracoscopic resection was performed in September 2024. Pathology confirmed viable metastatic neuroblastoma (poorly differentiated, low MKI). Crucially, IHC demonstrated Phox2b(+), MYCN(-), and C-MYC(-). An additional noteworthy finding was the marked increase in the Ki-67 labeling index. Compared with the post-induction surgical specimen, in which the Ki-67 index was approximately 8%, the resected residual lesion demonstrated a Ki-67 index of approximately 50%. Although the biological basis for this increase remains unclear, it suggests persistent proliferative activity within the residual tumor despite otherwise favorable systemic disease assessments.

### Follow-up and current status

The integration of GD2/GM-CSF/13-cis-RA maintenance followed by definitive surgical extirpation was followed by sustained remission. The latest contrast-enhanced MRI (April 2025) demonstrated slight paravertebral soft tissue thickening (T3-T9) but no abnormal enhancement, alongside normal thymic morphology. Currently, the patient is in regular, long-term follow-up and has been entirely off therapy for an extended period. Serial evaluations—including normal CBC, stable NSE, and normal hepatic/renal panels—confirm sustained remission (**Table 1**). The pediatric patient has successfully returned to school and remains in excellent overall health.

**Table 1:** Chronological timeline of clinical events, including presentation, laboratory findings, pathology, imaging, treatment, and follow-up.

Date/timepoint	Clinical event & intervention	Physical exam & key laboratory findings	Structured diagnostic assessment (imaging & pathology)
Mar 2023 (Age: 4y 11m)	Initial Presentation & Diagnosis	Physical Exam: Pallor, palpable abdominal mass Labs: NSE: 126 ng/ml; Dopamine: 4.79 nmol/L; Hb: 76 g/L.	PET/CT: Multi-site high metabolism (soft tissue, left kidney SUVmax 7.97, bone marrow SUVmax 34.33). Biopsy: Poorly differentiated NB. Ki-67: 80%. Genetics: 1p36 loss, 11q LOH. BM MRD: Bilateral positive.
Mar-Jun 2023	Induction Chemotherapy (Cycles 1-4)	Labs (May): NSE decreased to 27.8 ng/ml.	CT (May): 76% reduction in tumor volume. BM MRD (May): Right negative; Left positive (9.31%).
Jun 2023	Surgical Intervention (Tumor resection)	Clinical Status: Post-operative recovery.	Pathology: NB (low differentiation, low MKI). Ki-67 significantly dropped to ~8%. IHC: Phox2b(+), Syn(+), CD56(+).
Jul-Sep 2023	Consolidation Therapy & Radiotherapy (Cycles 5-6+RT)	Labs (Jul): NSE normalized to 11.9 ng/ml.	PET/CT (Aug): No obvious hypermetabolism (CR achieved). BM MRD: Bilateral negative.
Oct 2023-May 2024	Anti-GD2 Immunotherapy (Dinutuximab beta + GM-CSF + 13-cis-RA, 7 cycles)	Physical Exam: No palpable masses, transient fever, rash managed successfully] Labs: Routine blood and organ functions normal.	CT (Mar & Jul 2024): Suspicious enlarged thymus/soft tissue remnant (fluctuating size, 4.3×2.8 cm in Jul).
Aug-Sep 2024	The “Imaging Paradox” & Diagnostic Ambiguity	Clinical Status: Asymptomatic. Labs normal.	^123I-MIBG: Currie Score 0 (Metabolic CR/False Negative). ^68Ga-DOTA-NOC: Active T7 paravertebral nodule (0.5×0.5 cm), SSTR elevated (SUVmax 3.46/Positive).
Sep 2024	“Third-Look” Surgery (Thoracoscopic extirpation)	Clinical Status: Tolerated surgery well.	Pathology: Metastatic NB. IHC: Phox2b(+). Ki-67 surged to~50% (Phenotypic rebound).
Apr 2025-Present	Sustained Remission & Follow-up	Physical Exam: Healthy appearance, normal growth and development. Labs: NSE normal, CBC normal.	MRI (Apr 2025): T3-T9 paravertebral soft tissue slight thickening, no abnormal enhancement. Normal thymus. (Disease-free for >19 months)

## Discussion

The management of persistent soft tissue residual disease remains a challenging aspect of high-risk neuroblastoma (HR-NB) treatment, particularly in the era of anti-GD2 immunotherapy (5). In the present case, maintenance therapy with dinutuximab beta, GM-CSF, and 13-cis-retinoic acid was associated with sustained clearance of bone marrow minimal residual disease (MRD) and a complete metabolic response by conventional ^123I-MIBG assessment (6). Nevertheless, a persistent paravertebral soft tissue lesion remained detectable on structural imaging and was subsequently confirmed to contain viable neuroblastoma following surgical resection. This observation highlights the complexity of interpreting residual masses after multimodal therapy and underscores the importance of careful evaluation of persistent lesions despite otherwise favorable treatment responses (7).

A particularly notable feature of this case was the discordant functional imaging findings observed in a persistent residual lesion. Although the patient achieved a Curie score of 0 with no abnormal uptake on ^123I-MIBG scintigraphy, the unchanged paravertebral soft tissue mass demonstrated persistent tracer uptake on ^68Ga-DOTA-NOC PET/CT (8). This imaging discordance, in the context of otherwise favorable systemic response, prompted further evaluation with somatostatin receptor-targeted imaging, which ultimately contributed to the decision for surgical resection and was subsequently confirmed to represent viable neuroblastoma on histopathology. These findings suggest that negative ^123I-MIBG imaging does not necessarily exclude residual biologically active disease in selected patients and highlight the potential complementary role of ^68Ga-DOTA-NOC PET/CT when conventional imaging is inconclusive. In this context, multimodal functional imaging may provide additional value in the assessment of persistent soft tissue lesions that are difficult to characterize using standard response evaluation alone (9).

Another noteworthy observation was the marked increase in the Ki-67 labeling index, from approximately 8% in the post-induction surgical specimen to 50% in the lesion resected after immunotherapy. The biological significance of this finding remains uncertain. One possible explanation is that the residual lesion contained a subpopulation of tumor cells with distinct proliferative characteristics that became more prominent during treatment (10). Alternatively, differences in sampling, treatment-related changes in tumor composition, or other forms of biological heterogeneity may have contributed to the observed increase. Although the finding raises interesting biological questions, the available data do not permit definitive conclusions regarding the underlying mechanism.

The imaging discordance observed in this case may represent a macroscopic clinical manifestation of tumor lineage plasticity, a mechanism that has been well described in preclinical and translational studies of neuroblastoma. Under therapeutic selective pressure, neuroblastoma cells may undergo an adrenergic-to-mesenchymal transition (AMT), resulting in progressive loss of adrenergic features, including reduced expression of the norepinephrine transporter (NET) and potentially surface GD2 (11–13). From a clinical imaging perspective, such changes may be reflected as reduced or absent uptake on ^123I-MIBG scintigraphy despite the persistence of structurally identifiable lesions (14, 15). In contrast, residual somatostatin receptor (SSTR) expression may persist in a subset of tumor cells following phenotypic transition, which could account for the positive ^68Ga-DOTA-NOC PET/CT findings observed in this patient (16). In this context, the coexistence of MIBG negativity and DOTA-NOC positivity may be interpreted as an imaging-level expression of underlying tumor plasticity described in experimental models, rather than as evidence of complete biological inactivity of the lesion (9). In parallel, tumor microenvironmental factors may also contribute to the heterogeneous behavior of residual soft tissue lesions (17). Prior studies have shown that therapy-induced fibrosis, hypoxia, and extracellular matrix remodeling may alter tissue permeability and cellular interactions within residual tumor sites. In addition, immunosuppressive components of the microenvironment, including tumor-associated macrophages and mesenchymal stromal cells, may further modulate local immune effector activity and antibody-dependent cellular cytotoxicity (18). These factors together may help explain why residual lesions can persist in selected anatomical sites despite favorable systemic treatment response (19).

From a clinical perspective, this case has important implications for the management of persistent residual soft tissue disease. At present, there are no standardized guidelines specifically addressing the evaluation and treatment of residual masses that persist following anti-GD2 immunotherapy. Some residual lesions may represent fibrosis, necrosis, or treatment-induced maturation, whereas others may harbor viable tumor cells (20, 21). Consequently, management decisions often require integration of serial imaging findings, clinical context, and multidisciplinary assessment. In selected patients with persistent lesions and discordant imaging results, surgical resection may provide both definitive diagnosis and local disease control (21).

Importantly, the persistence of a residual lesion in our patient should not necessarily be interpreted as evidence of immunotherapy failure. Anti-GD2 immunotherapy likely contributed to sustained systemic disease control, including durable bone marrow remission and the absence of new metastatic lesions during follow-up. Rather than replacing systemic therapy, surgical intervention served as a complementary strategy for managing a localized residual focus that remained detectable despite otherwise favorable treatment responses. Although isolated reports of DOTA-positive/MIBG-negative neuroblastoma have been described, few reports have documented pathological confirmation of viable residual neuroblastoma following anti-GD2 immunotherapy in the setting of a Curie score of 0 and sustained bone marrow remission.

Several limitations should be acknowledged. This report describes a single patient and therefore cannot establish generalizable conclusions regarding imaging strategies or mechanisms of treatment resistance. Furthermore, no analyses of GD2 expression, norepinephrine transporter expression, tumor immune infiltrates, or molecular subtype were performed (22–24). As a result, mechanistic interpretations remain hypothesis-generating rather than definitive. Additional studies incorporating molecular characterization of residual lesions will be necessary to better understand the biological basis of discordant imaging findings and persistent soft tissue disease (25).

In conclusion, this case demonstrates that viable residual neuroblastoma may persist despite negative ^123I-MIBG findings and apparent systemic remission following anti-GD2 immunotherapy (26). For selected patients with persistent structural lesions, multimodal functional imaging may provide clinically useful information beyond conventional response assessment. When imaging findings remain discordant, carefully selected surgical intervention may contribute to both diagnostic clarification and durable local disease control.

## Conclusion

This case demonstrates that viable residual neuroblastoma may persist despite negative ^123I-MIBG findings and otherwise favorable systemic response following multimodal therapy and anti-GD2 immunotherapy. The discordance between ^123I-MIBG scintigraphy and ^68Ga-DOTA-NOC PET/CT highlights the potential limitations of relying on a single imaging modality when evaluating persistent soft tissue residual lesions.

In patients with persistent structural abnormalities and inconclusive conventional assessments, multimodal functional imaging may provide additional information regarding residual disease activity and assist clinical decision-making. In our patient, ^68Ga-DOTA-NOC PET/CT identified a residual lesion that was subsequently confirmed as viable neuroblastoma by surgical pathology, directly influencing further management.

Although the biological mechanisms underlying this imaging discordance remain uncertain, this case underscores the importance of integrating imaging findings with clinical context and histopathological confirmation when appropriate. The favorable outcome observed following surgical resection suggests that carefully selected local intervention may contribute to durable disease control in patients with persistent residual lesions.

Overall, this case supports a pragmatic management strategy combining systemic anti-GD2-based therapy, multimodal imaging assessment, and selective surgical intervention for clinically challenging soft tissue residual disease.

## Data Availability

The raw data supporting the conclusions of this article will be made available by the authors, without undue reservation.
